# Development of Functional Antibodies Directed to Human Dialyzable Leukocyte Extract (Transferon®)

**DOI:** 10.1155/2019/2754920

**Published:** 2019-05-16

**Authors:** Gabriela Mellado-Sánchez, Juan José Lázaro-Rodríguez, Sandra Avila, Luis Vallejo-Castillo, Said Vázquez-Leyva, Gregorio Carballo-Uicab, Marco Velasco-Velázquez, Emilio Medina-Rivero, Lenin Pavón, Rommel Chacón-Salinas, Sonia Mayra Pérez-Tapia

**Affiliations:** ^1^Unidad de Desarrollo e Investigación en Bioprocesos (UDIBI), Escuela Nacional de Ciencias Biológicas, Instituto Politécnico Nacional, CDMX, Mexico; ^2^Departamento de Farmacología, Centro de Investigación y de Estudios Avanzados del IPN (CINVESTAV-IPN), CDMX, Mexico; ^3^Departamento de Farmacología y Unidad Periférica de Investigación en Biomedicina Translacional (CMN 20 de noviembre, ISSSTE), Facultad de Medicina, Universidad Nacional Autónoma de México, Ciudad Universitaria, CDMX, Mexico; ^4^Laboratorio de Psicoinmunología, Instituto Nacional de Psiquiatría Ramón de la Fuente, CDMX, Mexico; ^5^Departamento de Inmunología, Escuela Nacional de Ciencias Biológicas, Instituto Politécnico Nacional, CDMX, Mexico

## Abstract

Transferon® is an immunomodulator made of a complex mixture of peptides from human dialyzable leucocyte extracts (hDLEs). Development of surrogate antibodies directed to hDLE is an indispensable tool for studies during process control and preclinical trials. These antibodies are fundamental for different analytical approaches, such as identity test and drug quantitation, as well as to characterize its pharmacokinetic and mechanisms of action. A previous murine study showed the inability of the peptides of Transferon® to induce antibody production by themselves; therefore, in this work, two approaches were tested to increase its immunogenicity: chemical conjugation of the peptides of Transferon® to carrier proteins and the use of a rabbit model. Bioconjugates were generated with Keyhole Limpet Hemocyanin (KLH) or Bovine Serum Albumin (BSA) through maleimide-activated carrier proteins. BALB/c mice and New Zealand rabbits were immunized with Transferon® conjugated to KLH or nonconjugated Transferon®. Animals that were immunized with conjugated Transferon® showed significant production of antibodies as evinced by the recognition of Transferon®-BSA conjugate in ELISA assays. Moreover, rabbits showed higher antibody titers when compared with mice. Neither mouse nor rabbits developed antibodies when immunized with nonconjugated Transferon®. Interestingly, rabbit antibodies were able to partially block IL-2 production in Jurkat cells after costimulation with Transferon®. In conclusion, it is feasible to elicit specific and functional antibodies anti-hDLE with different potential uses during the life cycle of the product.

## 1. Introduction

Transferon® is a hemoderivative used as biotherapeutic with immunomodulatory properties; its components are a complex mixture of peptides from human dialyzable leucocyte extracts (hDLEs); these peptides are constituted by different proportions of amino acids among them Gly, Glu, and Ala (almost 44% of the total content of proteinogenic amino acids) [1]. The safeness of a biotherapeutic is a mandatory requirement for its use in humans; in this regard, one of the nondesirable characteristics for this kind of products is the induction of antidrug antibodies (ADAs) that could diminish the effect of the drug or the formation of immunocomplexes that would lead to an inflammatory response. So far, there are no reports of ADA induction in patients treated with Transferon®, nor in mice [2], giving evidence of the safeness of this biotherapeutic along with null acute toxicity in mice (unpublished data).

To induce immunogenicity, biomolecules have to comply with some requirements: high molecular weight (above ~6-10 kDa), high structural complexity, certain level of degradability, and enough phylogenetic distance between two species (foreignness) [3]; therefore, peptides are not good immunogens; this could be the explanation of the negligible immunogenicity of Transferon®.

In spite of poor immunogenicity and enzymatic degradation sensibility, there are several advantages using peptides as immunogens, such as they can be produced, purified, and characterized easily; the immunogenicity can be specifically directed to known, relevant targets instead of undesirable virulence factors from pathogens in the antigen preparation, rendering in safer preparations with lower adverse effects in the host, no biosafety concerns for the researchers because they do not need to manage the whole virulent pathogen, there is no reversion leading to virulence and no risk of genetic integration or recombination, they can be produced on a large scale at a reasonable cost, and they are easy to store and transport [4].

Thus, several strategies to enhance peptides immunogenicity have been tested, including the design of multiepitope vaccines using different platforms for production and delivery (DNA vaccines, recombinant or synthetic peptides, dendrimers, nanoparticles, etc.) [5–9]; even for more than one pathogen [10], mixture with classical and novel adjuvants [11, 12], built-in vaccine adjuvants such as interleukins, TLR-agonists, or dendritic cell targeting [13–16], chemical conjugation to a carrier protein [4, 17, 18], or the use of different animal models instead of mice, like rabbits and chickens [19, 20] have been tested.

The development of surrogate antibodies to components present in Transferon® would be a useful tool for different studies such as process control and preclinical trials including tests such as identity, drug quantitation, and mainly to track molecules in pharmacokinetic studies in animal models to further characterization of this drug, mostly for the demonstration of mechanisms of action and adsorption.

In a previous study carried out by our group [2], we demonstrated the null immunogenicity of Transferon® in a murine model even though different immunization routes and adjuvants were employed, highlighting the safeness of the product but delaying the development of a surrogate antibody directed to this hDLE. Therefore, this work was aimed at inducing immunogenicity against this hemoderivative through two strategies; one of them was chemical conjugation of the Transferon® peptides to a carrier protein as the immunogen and the second one was to use the rabbit to achieve the induction of antibodies against peptides in general [21]. The resultant antibodies would have different potential purposes during the life cycle of this hemoderivative.

## 2. Materials and Methods

### 2.1. Animals

BALB/c mice (male, 6-8 weeks of age, 15-20 g) were purchased from Ferandelh (Mexico City, Mexico). All mice were housed in a P/NC IVC system (Allentown Inc., Allentown, NJ) at the Unidad de Desarrollo e Investigación en Bioprocesos (UDIBI) animal facility maintained at 18 - 24°C with a 40 - 70% relative humidity and a 12-hour light/dark cycle. For feeding, mice had free access to sterilized Teklad global 18% (Envigo, Franklin, NJ) and filtered water, all the time. New Zealand rabbits (female, 3-6 months of age) were obtained from the Escuela Nacional de Ciencias Biológicas (ENCB) animal facility; they were housed in standard conditions of temperature, humidity, and light. All experimental procedures with animals were performed according to the Mexican guideline and the International Guide for the Care and Use of Laboratory Animals [22, 23]. These experimental procedures were approved by the CIPFT research committee from UDIMEB and by the CEI Ethical and Research Committee from ENCB under respective codes (for mice protocol FTU/P2/17/012-PRO and CEI-ENCB-013-2017 and for rabbits FTU/P2/17/016-PRO and CEI-ENCB-022-2017). All efforts were made to minimize animal suffering and reduce the number of animals used.

### 2.2. Conjugation of Transferon® Peptides with Carrier Proteins

The Transferon® dried-freeze batch 17G15 was used. This batch complied with all quality control tests such as endotoxin content, microbiological burden, total protein concentration, physicochemical characterization, and biological potency *in vitro* as described previously [1].

Given that maleimide-activated carrier proteins were used to conjugation process, these are bound to cysteines only; thus, the resultant bioconjugate is composed of Transferon® cysteine-rich peptides (TCRP), which were covalently conjugated to Keyhole Limpet Hemocyanin (TCRP-KLH) and to Bovine Serum Albumin (TCRP-BSA). The KLH conjugation kit was purchased from Sigma-Aldrich (Darmstadt, Germany); meanwhile, maleimide-activated BSA was obtained from Innova Biosciences (Cambridge, UK). Briefly, activated carrier proteins were reconstituted with sodium phosphate buffer and immediately mixed with reconstituted Transferon® in water; the mixture was degassed for 10 minutes while stirring; further incubation with no degasification was performed for extra two hours at room temperature. Conjugates were aliquoted and kept at -70°C until use. Conjugation efficiency was evaluated measuring residual cysteines through a calibration curve made of a cysteine standard.

### 2.3. Immunization Scheme

TCRP-KLH conjugate and Transferon® alone were used as immunogens; these were mixed 1 : 1 *v*/*v* with Incomplete Freund's Adjuvant (IFA) (Sigma, St. Louis, MO, USA).

**Figure 1:**
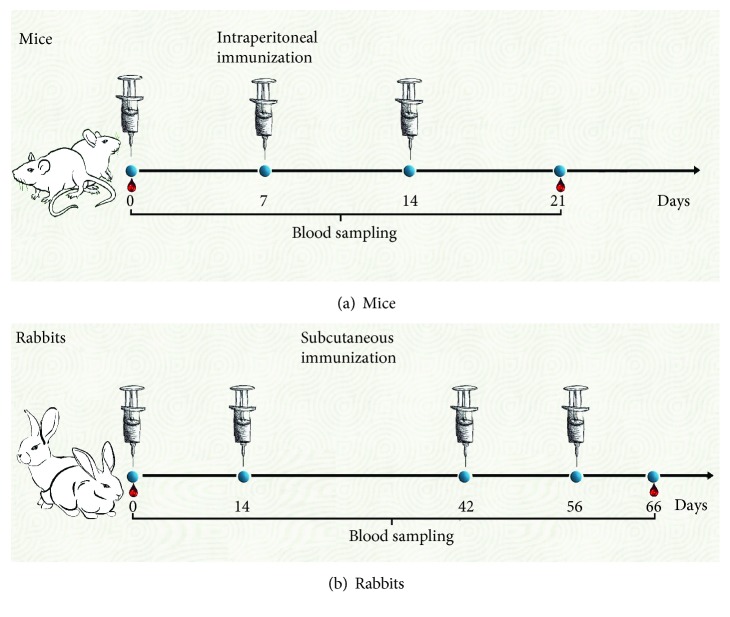
Immunization schemes for animals. TCRP-KLH and Transferon®, both admixed with IFA, were used as immunogens. (a) Mice received intraperitoneal administrations and (b) rabbits received subcutaneous immunizations. Blood samples were collected both from preimmune and immune animals.

For mice, immunization scheme comprised three intraperitoneal immunizations (100 *μ*g protein/dose/mouse) to groups of 6 mice. Blood samples were collected from the facial vein into microtainer tube blood sampling (Axygen^TM^, Union City, CA, USA). Serum samples were obtained by centrifugation (600 *g*, 15 min) and stored at -70°C until analysis (Figure 1(a)).

As a proof of concept, one rabbit was immunized with TCRP-KLH and another one with Transferon® alone.  Figure 1(b) depicts the immunization protocol formed by four subcutaneous immunizations on the back (250 *μ*g protein/dose/rabbit), and blood samples' collection from the ear vein was obtained; serum was processed in the same way described before.

Samples from a previous study were tested for reactivity against TCRP-KLH antigen [2] (Supplemental Figure).

### 2.4. Determination of Antibody Response against Transferon® Peptides by Indirect ELISA

Specific antibodies against TCRP-KLH, TCRP-BSA, KLH, and BSA were measured in serum from immunized animals.

For indirect ELISA assays, wells of 96-well MaxiSorp ELISA plates (Nunc, Roskilde, Denmark) were coated with 50 *μ*L of the corresponding antigen (bioconjugates or proteins alone) at 0.25 *μ*g/mL [for antigen titration assay (see section 3.1.1), 0.0025 to 250 *μ*g/mL of TCRP-KLH was used] diluted in carbonate buffer pH 9.5 from BD OptEIA™ Reagent Set B (BD Biosciences, Franklin Lakes, NY, USA), and they were incubated overnight at 4°C. From all ELISA techniques described in this work, between one step to another, the plate was washed 3 times with 1X wash buffer from BD OptEIA™ Reagent Set B and incubation steps were performed during 1 h at 37°C unless otherwise be stated. The plates were blocked with 100 *μ*L of 5% skim milk (Carnation Clavel, Nestlé, Mexico) in diluent buffer (blocking solution) and incubated. Serum dilutions from different animals were prepared in blocking solution, 50 *μ*L of each one was placed on respective wells and incubated. Then, 50 *μ*L of anti-mouse IgG-HRP (Sigma) diluted 1 : 5,000 or anti-rabbit IgG-HRP from Santa Cruz Biotechnology (Dallas, TX, USA) diluted 1 : 10,000 was added to each well, and the plates were incubated and washed as usual. The detection of specific antibodies was achieved by the addition of peroxidase substrate mixed with the chromogen from BD OptEIA™ Reagent Set B. The colorimetric reaction was halted after 20 min at RT in darkness by the addition of 30 *μ*L stop solution from BD OptEIA™ Reagent Set B to each well. The optical density (OD) values at 450 nm/570 nm were measured in an EPOCH plate reader (BioTek®, Winooski, VT, USA).

### 2.5. Purification of Rabbit IgGs with Protein-A

Serum from the rabbit immunized with TCRP-KLH was purified through affinity chromatography using a stationary phase made of protein-A (MabSelect SuRe resin) (GE Healthcare®, Chicago, IL, USA). Briefly, the resin was packed into an XK 16/20 column (GE Healthcare®), which was coupled to a UV detector as a part of the FPLC equipment (Akta-GE Healthcare). Rabbit serum diluted 1 : 2 in PBS was completely infused into the PBS-equilibrated protein-A column. Protein A-column was washed using 10 volumes of PBS to elute proteins that were weakly attached to the stationary phase. Then, rabbit IgG captured on protein-A was eluted by infusing 0.1 M acetic acid pH 2.8 buffer. Elution buffer of purified IgGs was changed to PBS pH 7.4 with an ultrafiltration device Amicon 30 kDa (Millipore, Darmstadt, Germany); IgGs were sterilized by 0.2 *μ*m Millex filtration unit (Millipore), aliquoted and kept at 4°C until use.

### 2.6. Transferon® Peptide Detection by a Capture/Sandwich ELISA

**Figure 2:**
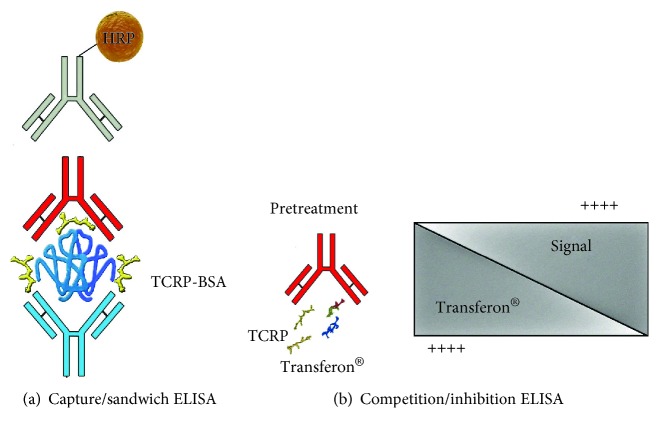
ELISA techniques. Capture/sandwich ELISA and competition/inhibition ELISA. Different antibodies used, in blue: rabbit IgG anti-TCRP-KLH; in red: mice sera pool anti-TCRP-KLH; in grey: HRP-labeled anti-mouse IgG.

ELISA plates were coated with 50 *μ*L of the corresponding concentration of purified rabbit IgG antibodies diluted in carbonate buffer pH 9.5. Plates were incubated for 3 hours at 37°C. Afterward, wells were blocked with 100 *μ*L of blocking solution and incubated overnight at 4°C. Next, 50 *μ*L of TCRP-BSA (0.01, 0.05, 0.25, and 1.25 *μ*g/mL) was added, and the plates were incubated. Then, 50 *μ*L of pooled mice sera anti-TCRP-KLH diluted 1 : 100 was added. The next steps were executed as described above, and the whole system is represented at Figure 2(a).

### 2.7. Transferon® Peptide Detection by a Competition/Inhibition ELISA

This type of ELISA was performed similarly at capture ELISA, except for the plates that were incubated for 4 hours at 37°C with the capture antibody and with an additional step that allowed the interaction of Transferon® peptides with pooled sera of hyperimmune mice (anti-TCRP-KLH). Each mixture was prepared to contain different concentrations of Transferon® and pooled mice sera anti-TCRP-KLH diluted in the mixture 1 : 100; meanwhile, 0.25 *μ*g/mL of TCRP-BSA was added to the plate. Both the plate and the mixtures were incubated simultaneously for 1 hour at 37°C. After this time, 50 *μ*L of the mixtures was added to the corresponding wells on the plate, and the incubation proceeded as usual. The rest of the procedure was performed as described in the previous paragraph; a schematic depiction of the inhibition and its effect is shown in Figure 2(b).

### 2.8. Functional Assay of IgG Anti-TCRP

#### 2.8.1. Cell Culture and Costimulation

Jurkat clone E6-1 cells were grown in RPMI-1640 medium from ATCC (Manassas, VA, USA) supplemented with 10% FBS (Gibco, Grand Island, NY, USA) with an atmosphere of 5% of CO_2_ at 37°C; cells were deprived of FBS by 12 h in the same incubation conditions; after that, RPMI-1640 medium was changed to AIM-V medium (ThermoFisher Scientific, Grand Island, NY, USA); the cells were counted; viable cells were adjusted at 5 × 10^5^ cell/mL. A mixture of 5,000 *μ*g/mL of Transferon® with 33.3 *μ*g/mL of rabbit IgG anti-TCRP-KLH antibodies was incubated overnight at 4°C and then ultrafiltrated through a 10 kDa membrane (Millipore). This ultrafiltrated mixture was used to costimulate 5 × 10^4^cell/well in a 200 *μ*L of total volume as final concentrations of 12.5 *μ*g/mL of concanavalin A (Sigma-Aldrich). Corresponding incubation controls were tested: incubation control (PBS), Transferon®, and anti-TCRP-KLH antibodies. Cells with different treatments were further incubated for 72 h with 5% of CO_2_ at 37°C. Plates were centrifuged at 1,000 rpm at 25°C to pellet the cells, and supernatants were collected and kept at -70°C until use.

#### 2.8.2. Human IL-2 (hIL-2) Quantitation

The determination of hIL-2 in the culture supernatants was performed accordingly to the manufacturer's instructions (Human IL-2 ELISA Set, BD Biosciences, San Diego, CA, USA).

### 2.9. Statistical Analysis

Statistical analysis was executed using the GraphPad® Software (GraphPad Software, La Jolla, California, USA, http://www.graphpad.com). Paired *t*-test was performed for the most of ELISA experiments, except for capture/sandwich ELISA results which were analyzed with two-way ANOVA, whereas one-way ANOVA was used for competition/inhibition ELISA and functional assay experiment. In all cases, the mean ± standard deviation (SD) is reported. Statistical significance is described using these signs: ^∗^ *P* ≤ 0.05; ^∗∗^ *P* ≤ 0.01.

## 3. Results

### 3.1. Determination of Antibody Response against Transferon® Peptides by Indirect ELISA

#### 3.1.1. Optimization of the System

**Figure 3:**
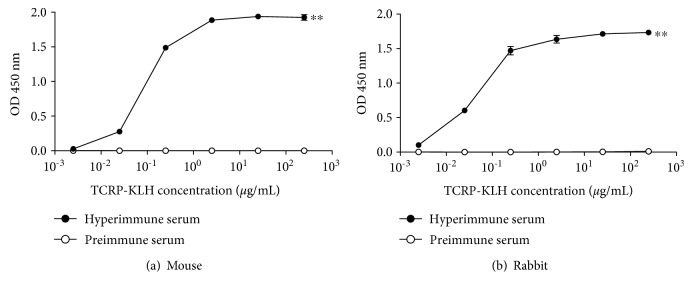
Evaluation of anti-TRCP-KLH antibodies and titration of antigen from mouse and rabbit sera. Before and after immunization protocols (see Figure 1), blood samples were taken to detect specific IgG anti-TCRP-KLH antibodies in the serum of animals immunized with the bioconjugate. An indirect ELISA was performed sensitizing plates from 2.5 × 10^−3^ to 2.5 × 10^2^ *μ* g/mL of TCRP-KLH. (a) A pool of mice sera was diluted 1 : 1,000. (b) Serum from rabbit was diluted 1 : 10,000. Technical triplicates were carried out. ^∗∗^ *P* ≤ 0.01.

Both mouse and rabbit generated strong antibody to KLH, showing their high immunogenic property, as expected (Figure 3). Additionally, this experiment was used to select the concentration of antigen; after this assay, the 0.25 *μ*g/mL of TCRP-KLH was established as the antigen concentration for the rest of the experiments. This was chosen because it rendered the highest signal which still behaves in direct proportion regarding antigen concentration; higher concentrations showed the saturation of the system seen as an asymptote. However, in this assay, it was not possible to discriminate antibodies directed to the carrier protein and those against TCRP.

#### 3.1.2. Detection of Anti-TCRP Antibodies

**Figure 4:**
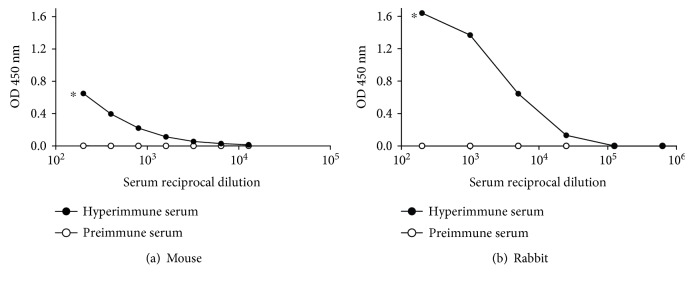
Antibody responses in mouse and rabbit sera against TCRP. An indirect ELISA was performed sensitizing plates 0.25 *μ*g/mL of TCRP-BSA. (a) A pool of mice sera was twofold diluted ranging from 1 : 200 to 1 : 12,800. (b) Serum from rabbit was fivefold diluted ranging from 1 : 200 to 1 : 625,000. Technical triplicates were carried out. ^∗^ *P* ≤ 0.05.

In order to detect antibodies only targeted to TCRP, in the ELISA assay, we used the bioconjugate TCRP-BSA to sensitize ELISA plate wells. We noticed that both mouse and rabbit produced anti-TCRP antibodies, with higher antibody titers in rabbit (Figure 4).

**Figure 5:**
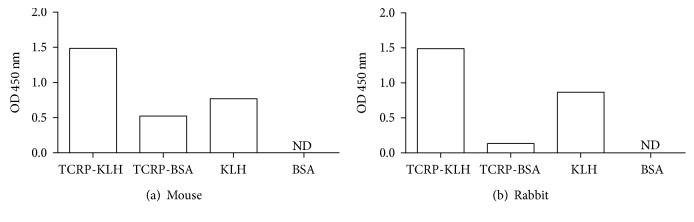
Antibody responses in mouse and rabbit sera against different antigens. Different antigens were used for the sensitization step in an indirect ELISA (TCRP-KLH, TCRP-BSA, KLH, and BSA) at 0.25 *μ*g/mL. (a) A pool of mice sera was diluted 1 : 1,000. (b) Serum from rabbit was diluted 1 : 25,000. ND: nondetected. Technical duplicates were carried out.

To compare the antibody response to each molecule that constitutes the bioconjugates used in this work, we employed the same serum dilution and the four antigens: TCRP-KLH, TCRP-BSA, KLH, and BSA (Figure 5). The highest response was against TCRP-KLH owing to the sum of responses against the two components, then the second main response was seen towards the carrier protein KLH; an evident signal was detected to TCRP-BSA, but no signal was detected to BSA, meaning that antibodies were generated against TCRP peptides conjugated to BSA (Figure 5). Furthermore, these two assays (Figures 4 and 5) showed that rabbit induced a stronger antibody production against peptides since higher serum dilutions rendered similar OD signals regarding those obtained with mice.

#### 3.1.3. Effect of the Chemical Conjugation over the Immunogenicity of Transferon®

**Figure 6:**
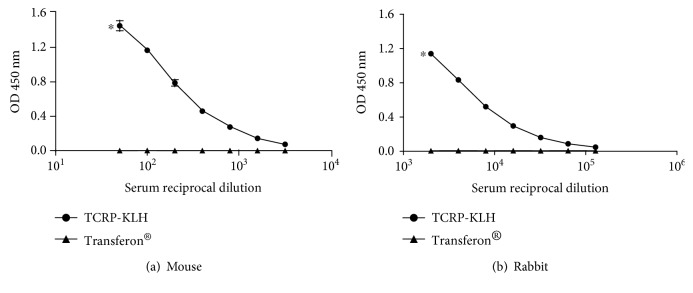
Comparison of Transferon® immunogenicity in animals inoculated with TCRP-KLH or Transferon® nonconjugated. An indirect ELISA was performed sensitizing plates 0.25 *μ*g/mL of TCRP-BSA. (a) A pool of mice sera was twofold diluted ranging from 1 : 50 to 1 : 3,200. (b) Serum from rabbit was fivefold diluted ranging from 1 : 200 to 1 : 625,000. Technical triplicates were carried out. ^∗^ *P* ≤ 0.5.

Next, we evaluated the response of mice and rabbits to unconjugated TCRP peptides and compared with the antibody production when immunized with TCRP-KLH. As shown in Figure 6, no response was achieved in whichever animal model when Transferon® was used alone as the immunogen; anti-TCRP antibodies were induced only in animals that were immunized with the bioconjugate TCRP-KLH.

These results are in agreement with those obtained in our previous study [2], where no antibodies were detected in mice immunized with Transferon® and different adjuvants. But a question arose, the influence of adjuvant over the response obtained with unconjugated peptides of Transferon®, given that in the preliminary study two extra adjuvants were analyzed (Al(OH)_3_ and TiterMax® Gold®), we reanalyzed those sera using the bioconjugates as antigens, searching for a signal; nonetheless, this was negligible (Supplemental Figure) in spite of using a more sensible system through the conjugation of Transferon® peptides. As proof of the system performance, a positive pool of sera was added obtaining evident responses.

### 3.2. Transferon® Peptide Detection by a Capture/Sandwich ELISA

**Figure 7:**
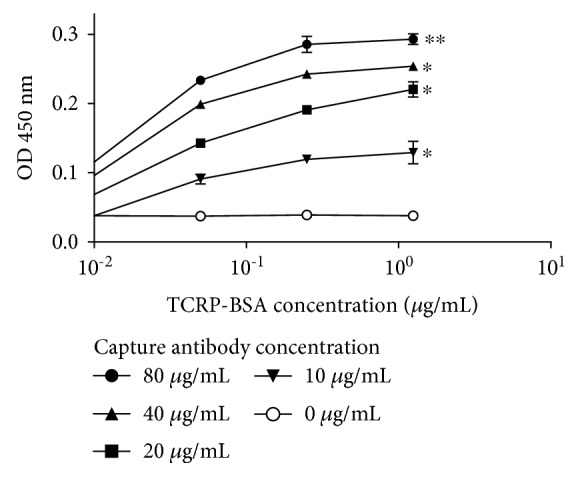
Effect of the capture antibody concentration in a sandwich ELISA. Rabbit IgG anti-TCRP-KLH was purified from hyperimmune serum through protein-A column, then it was tested at different concentration for sensitizing the ELISA plates; TCRP-BSA was used as antigen with fivefold increments ranging 0.01-1.25 *μ*g/mL; the pool of mice was diluted 1 : 100. Full system depiction is shown in Figure 2. Technical triplicates were carried out. ^∗^ *P* ≤ 0.05, ^∗∗^ *P* ≤ 0.01.

Due to the reason that both animal models had elicited anti-TCRP antibodies, we used useful tools to develop a capture ELISA since this technique displays a higher sensitivity compared with indirect ELISA; the design is depicted in Figure 2; previously, rabbit serum was purified. As can be seen in Figure 7, a concentration-response effect was achieved to be able to detect up to 0.01 *μ*g/mL of antigen. Nevertheless, the OD responses were low despite to have concentrated the rabbit serum. With this experiment, it was established that antigen concentration (TCRP-BSA) should be 0.25 *μ*g/mL owing that the results for the next concentration (1.25 *μ*g/mL) were very close.

### 3.3. Transferon® Peptide Detection by a Competition/Inhibition ELISA

**Figure 8:**
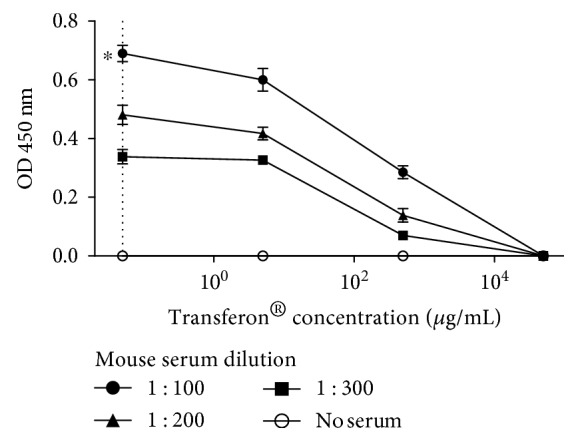
Detection of Transferon® peptides through a competition ELISA. The system used is depicted in Figure 2. The capture antibody (rabbit purified IgG anti-TCRP-KLH) was used at 80 *μ*g/mL; meanwhile, antigen concentration (TCRP-BSA) was 0.25 *μ*g/mL. Different concentrations of Transferon® (5, 500, and 50,000 *μ*g/mL) or diluent (dotted line) were preincubated with three dilutions of the secondary antibody (pool of mice sera). Technical triplicates were carried out. ^∗^ *P* ≤ 0.05.

To evaluate whether our ELISA system could detect free peptides from Transferon®, we designed a competition ELISA. In this system, Transferon® was used as a competitor of TCRP-BSA (antigen). The results of this assay are shown in Figure 8, where a clear diminution of the OD is achieved depending on the concentration of unconjugated Transferon®.

### 3.4. Functional Assay of IgG Anti-TCRP

**Figure 9:**
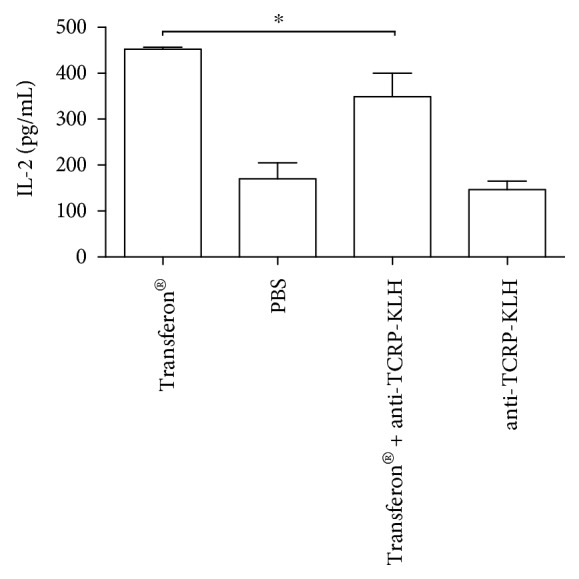
Effect of preincubation with anti-TCRP-KLH over the biological activity of Transferon®. Transferon® was preincubated with the rabbit purified IgG anti-TCPR-KLH, and the ultrafiltrated fraction (<10 kDa) was evaluated in IL-2 induction model in Jurkat cells. Experimental triplicates were carried out for a representative experiment. ^∗^ *P* ≤ 0.05.

Transferon®'s biological activity has been recently evaluated using an IL-2 induction *in vitro* model (unpublished data). This model was used to determine if the active peptide fraction of Transferon® is recognized by the rabbit IgG anti-TCRP-KLH. As shown in Figure 9, the ultrafiltrated fraction of Transferon® preincubated with anti-TCRP-KLH antibodies evinced a significant decrement respect to the whole Transferon® treatment.

## 4. Discussion

Transferon® is a biotherapeutic made of hDLE; the production of this medicament is carried out at the Pharma-FT, a division of the Unidad de Investigación, Desarrollo e Innovación Médica y Biotecnológica (UDIMEB); this follows the applicable Mexican legislation for hemoderivatives NOM-253-SSA1-2012 [24], and it is produced under Good Manufacturing Practices established at NOM-059-SSA1-2013 [25]. During Transferon®'s production process exists different critical points in which an identity test of the product or its quantitation through an immunoassay could be helpful for complementary tests to the current quality assessment. The key components to achieving the development of these assays are surrogate antibodies anti-Transferon® due to their high specificity. Moreover, these antibodies could be excellent tools during the pharmacokinetics studies of the product and other potential applications.

The main challenge to induce an immune response towards the components of Transferon®, in other words, against peptides from human leukocytes, is to induce an immune response directed to a huge diversity of peptides between 0.2-10 kDa [26] and low structural complexity. Because Transferon® has shown low immunogenicity in a murine model [2], in order to develop surrogate antibodies against Transferon®, two approaches were explored: conjugation of Transferon®' peptides to highly immunogenic protein and the use of an animal model that produce antibodies to free peptides.

A hapten is a small organic molecule that is antigenic but not immunogenic unless it is covalently coupled to a carrier protein [3]. One of the most used carriers for immunization assays in mammals is KLH because it is multimeric, it is expressed as two subunit isoforms of 350-400 kDa, and it comes from a molluscan (long phylogenetic distance to mammals); all of these are the requirements for a good immunogen. This strategy to transform a low immunogenic molecule into an immunogenic one has been successfully used for a variety of small molecules, among them, peptides. Our results show, in fact, some Transferon®'s peptides have haptenic properties, since antibodies were induced. However, ought to chemical conjugation of peptides was carried out through maleimide-preactivated proteins (KLH and BSA); only peptides that contain the target amino acid residue cysteine were bound to the carrier proteins. This amino acid residue constitutes approximately 3% of relative average abundance out of the total of Transferon® peptides [1], and more recently, it has been determined that with the total peptides that are present in Transferon®, only 20% contain at least one cysteine residue (TCRP) (unpublished data). One of the limitations of the study is that only a fraction of the total amount of peptides was conjugated to KLH, the carrier protein; therefore, the total immunogenicity of hDLE is underestimated. This can be overcome testing another chemical conjugation reagent towards other amino acid residues different from cysteines.

Despite the advantages of using hapten-carrier protein as immunogens, these have some issues as well, among them are the following: the background of the immune response towards carrier proteins, the irregular arrangement of peptides on the protein, and the neoantigen formation depending on the conjugation method [4]. We had to face two of the mentioned disadvantages; the first was overcome using a heterologous antigen in the ELISA plates, TCRP-BSA bioconjugate; to get to know what was the real input of the TCRP over the total immunogenicity measured, we subtracted the antibody response against the carrier protein of the total antibody response generated. The second issue was the possibility of that observed antibody responses was directed to new epitopes formed because of the conjugation method; to discard this, additional modalities of ELISA were performed.

To demonstrate undoubtedly the direct binding of some Transferon®'s peptides to the rabbit and mouse antibodies, capture ELISA and competition/inhibition ELISA systems were developed. Specifically in the competition/inhibition ELISA system, our results indicated that once the antigen-binding site of the antibodies from mice was occupied for Transferon®'s peptides, these antibodies could not attach anymore to the bioconjugate attached to the plate (TCRP-BSA) through purified rabbit IgG; this effect was also dependent on the concentration of the pool of mice sera. It was necessary to use high concentrations of Transferon® to inhibit the total signal; this phenomenon probably means that only a few portions of peptides of out of the total composition induced antibodies or despite the fact of enough levels of antibodies, these were directed to a poor variety of peptides of the whole composition of the biotherapeutic.

As proof of concept, we analyzed the feasibility of using a different animal model as an alternative to increasing immunogenicity of peptides. It has been reported that rabbits given their intrinsic hypervariability in the production of antibodies, they have the capability of reordering the light and heavy chains in a unique way, possibly enhancing immune responses against poor immunogens [19, 27], such as unconjugated peptides [21, 28]. Our results may lead to having evidence that effectively, the rabbit is a better model than the mouse to induce anti-hDLE antibodies due to higher antibody levels detected, although the responses seen in mice meant the covalent conjugation was more important than the animal model to achieve this.

Regarding functional assay, rabbit polyclonal IgG antibodies were chosen over mouse system due to the amount of serum and the use of purified rabbit IgG anti-TCRP-KLH antibodies to avoid the background of the rest of the seric proteins. A partial decreasing of IL-2 production in Jurkat cells was obtained; this result suggests that active peptides of Transferon® are among the molecules recognized by the rabbit polyclonal antibody generated in this work. Probably when we achieved to develop a wider spectrum of antibodies anti-Transferon®'s peptides, this decrement could be total if all active peptides of this hemoderivative were detected by this biological activity model.

Still, it is necessary to augment the number of rabbits to test the reproducibility of the observed phenomena and take into account that there were differences between immunization protocols (dose, site of immunization, and the period of time among administrations). Hence, more studies are necessary to optimize the responses in mice that despite the minimal amount of serum in comparison with the rabbit, a more manageable model and mouse monoclonal antibody technologies are more common in labs [28].

At this point, it is unknown what are the molecular targets of the elicited antibodies; further studies are needed to discover this, along with sequencing analysis that is currently carried out by our group in this hemoderivative.

To the best of our knowledge, this is the first time that the development of antibody anti-hDLE is reported, which it could be used as a useful tool for the pharmacokinetics of this biotherapeutic, identity proof to avoid falsification hDLE, and as an alternative methodology to quantify hDLE, among others.

## 5. Conclusions

Some of Transferon®'s peptides have haptenic characteristics when they are conjugated chemically to a carrier protein, eliciting a discrete but evident, specific, and functional antibody response directed to TCRP. The chemical conjugation was a more effective approach than the change of animal model in the induction of antibody responses against the hDLE; additionally, the rabbit was a better animal model than the mouse. It is possible to induce antibody anti-hDLE with several potential uses during the life cycle of the product.

## Data Availability

The data used to support the findings of this study are available from the corresponding author upon request.

## Supplementary Materials

Supplementary Materials Supplemental Figure: shows no adjuvant influence over the induction of antibodies when animals were immunized with Transferon® alone. Corroboration of no ADAS anti-Transferon® induction in a murine model. An indirect ELISA was performed sensitizing with 0.25 *μ*g/mL of antigens. (A) TCRP-KLH was used as antigen, and mouse pool sera anti-TCRP-KLH was diluted 1 : 16,000. (B) TCRP-BSA was used as antigen, and mouse pool sera anti-TCRP-KLH was diluted 1 : 200. Sera from the previous study were diluted 1 : 50 to test their reactivity to both antigens. Technical duplicates were carried out. ND: Nondetected.
