# Supplementary figures and images for: Development of Functional Antibodies Directed to Human Dialyzable Leukocyte Extract (Transferon®)
Source: J Immunol Res. 2019 May 16;2019:2754920. doi: 10.1155/2019/2754920 (PMC6541944; doi:10.1155/2019/2754920)

## Supplemental Figure 1

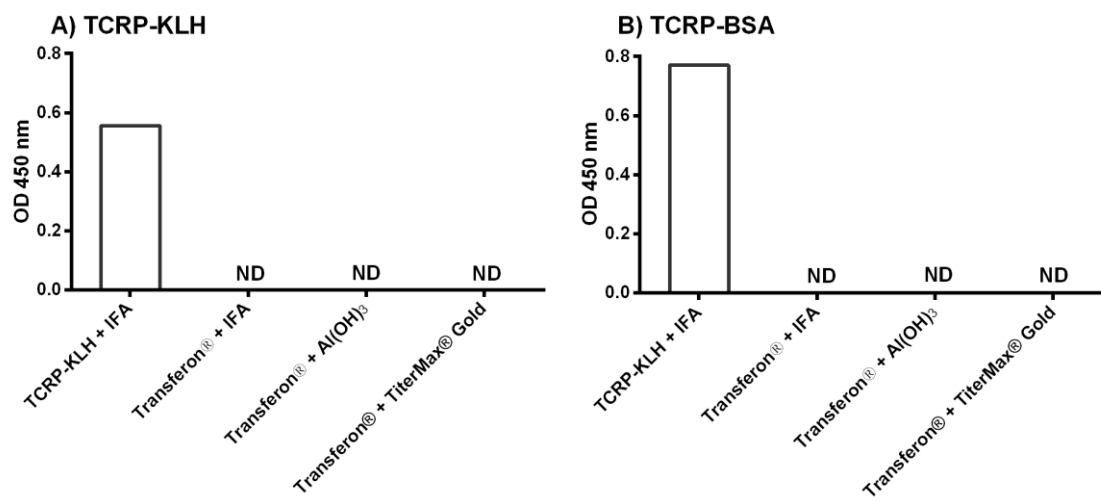

Supplement: Supplementary Materials — Supplemental Figure: shows no adjuvant influence over the induction of antibodies when animals were immunized with Transferon® alone. Corroboration of no ADAS anti-Transferon® induction in a murine model. An indirect ELISA was performed sensitizing with 0.25 μg/mL of antigens. (A) TCRP-KLH was used as antigen, and mouse pool sera anti-TCRP-KLH was diluted 1 : 16,000. (B) TCRP-BSA was used as antigen, and mouse pool sera anti-TCRP-KLH was diluted 1 : 200. Sera from the previous study were diluted 1 : 50 to test their reactivity to both antigens. Technical duplicates were carried out. ND: Nondetected. [file 2754920.f1.pdf]
